# Ultrasound-Based Wearable for Older Chronic Back Pain Patients: A Requirement Analysis of a User Interface for Biofeedback

**DOI:** 10.3390/geriatrics11030059

**Published:** 2026-05-15

**Authors:** Luis Perotti, Oskar Stamm, Susan Vorwerg-Gall, Lisa Mesletzky, Drin Ferizaj, Steffen Dißmann, Sandra Stube-Lahmann, Marc Fournelle, Nils Lahmann, Ursula Müller-Werdan

**Affiliations:** 1 Department of Geriatrics and Medical Gerontology, Charité—Universitätsmedizin Berlin, Corporate Member of Freie Universität Berlin and Humboldt-Universität zu Berlin, 13347 Berlin, Germanydrin.ferizaj@charite.de (D.F.); ursula.mueller-werdan@charite.de (U.M.-W.); 2 Department of Ultrasound, Fraunhofer Institute for Biomedical Engineering, 66280 Sulzbach, Germany

**Keywords:** chronic low back pain, older adults, ultrasound biofeedback, user interface design, gamification in rehabilitation

## Abstract

Purpose: This study explores how older adults with chronic back pain (CBP) evaluate different user interface (UI) designs and gamification elements for an ultrasound-based wearable providing real-time biofeedback during segmental stabilization exercises (SSE). The aim is to identify design preferences and motivational factors to enhance usability, engagement, and adherence in this specific population. Methods: We conducted a mixed-methods study with 15 older adults (aged ≥ 65) experiencing CBP. Participants interacted with three UI mockups (simple, anatomical, and playful) via a Wizard-of-Oz simulation and evaluated additional motivational elements (e.g., points, badges, progress charts). Semi-structured interviews and the Technology Usage Inventory (TUI) subscales were used to assess usability, acceptance, and intention to use. Results: Participants preferred the simple and anatomical UI designs, citing clarity, professionalism, and ease of interpretation. The playful design was viewed as less appropriate due to perceived infantilization. Game elements such as progress tracking, points, and levels were positively received, while competitive features like leaderboards were viewed critically. Most participants expressed interest in integrating pain education, favoring multimedia formats. Conclusions: Digital health tools for older adults must prioritize intuitive, medically reliable interfaces and allow personalization of motivational and educational components. The findings highlight the need for age-appropriate UI design and suggest that well-balanced gamification and educational features may enhance perceived acceptance and have the potential to support long-term use, which should be evaluated in longitudinal studies.

## 1. Introduction

Chronic back pain represents one of the most prevalent health conditions in Germany, with 61.3% of people reporting back pain within the last twelve months. The condition is particularly common among older adults, with 33.9% of women and 25.9% of men suffering from chronic back pain (defined as pain lasting longer than three months). This condition significantly reduces the quality of life in elderly populations and generates substantial healthcare costs due to treatments and work absenteeism [1].

Segmental stabilization exercises (SSE) are a widely used physiotherapeutic approach for treating chronic back pain. These exercises, also termed core, spinal, or trunk stability exercises, aim to improve active core stability by targeting deep muscles including the m. transversus abdominis (deepest abdominal muscle), mm. multifidii (small back muscles connecting individual vertebrae), diaphragm, and pelvic floor muscles [2]. Patients with chronic back pain typically have reduced local stability and strength of these deep muscles, with atrophy, earlier fatigue, and delayed contraction of the transversus abdominis muscle [3].

Through SSE, patients learn to control these deep core muscles to reduce back pain by creating a muscular “corset” that supports the spine and protects joints from compressive forces [4]. A common SSE technique is the abdominal drawing-in maneuver, which allows for isolated contraction of the transverse abdominal muscle (TrA) [5]. This exercise is initially challenging because the superficial and deep muscles are usually exercised simultaneously, yet only minimal contraction of deep muscles is required for optimal stabilization.

The effectiveness of SSE for chronic back pain is well-established. Coulumbe et al. [6] demonstrated in their meta-analysis that core stability exercises are more effective in reducing pain and improving functional status compared to conventional physical therapy in patients with chronic low back pain. However, performing SSE correctly remains challenging for both physiotherapists and patients, as it requires significant concentration and body awareness to achieve the right level of contraction in deep muscles without activating superficial muscles [7].

Learning proper SSE techniques poses a significant challenge due to the nature of deep muscle contractions, which do not produce visible movements but are crucial for restoring spinal stability at the segmental level. Effective training necessitates precise feedback on the function of these deep muscles, particularly during the initial learning phases. Traditional feedback methods often involve physical therapists palpating or tapping over patients’ abdominal muscles and providing sensory and verbal cues. Alternatively, patients may attempt to palpate their own abdominal muscles during exercises. However, these methods are subjective and may not reliably indicate accurate deep muscle activation. In a study by Valentín-Mazarracín et al. [8], the reliability and concurrent validity of manual palpation during the abdominal drawing-in maneuver were examined. The results showed good to excellent intra- and inter-rater reliability of palpation, as well as acceptable concurrent validity when compared with rehabilitative ultrasound imaging in identifying preferred activation of the deep abdominal muscles, particularly the transversus abdominis. These findings support the use of palpation in clinical practice, although it remains subjective and may not offer the same level of accuracy as imaging-based techniques.

Rehabilitative ultrasound imaging (RUSI) has emerged as a valuable biofeedback tool to improve motor performance and learning of trunk muscles in individuals with low back pain. Ultrasound feedback has been shown to effectively facilitate activation of the transversus abdominis during an abdominal drawing-in maneuver and the lumbar multifidus during isometric contraction [9]. Lin et al. [10] found that the thickness of deep abdominal muscles increased when performing stabilization exercises with ultrasound guidance. Real-time ultrasound feedback can significantly reduce the number of trials needed to learn proper muscle activation techniques [11]. However, several practical constraints impede widespread implementation. Ultrasound images of deep abdominal and back muscles are notoriously difficult to interpret, requiring experienced and highly specialized professionals for safe interpretation and to avoid low intra-observer reliability [7]. Furthermore, only practitioners with specialized training can effectively analyze ultrasound images of deep core muscles, limiting accessibility [12]. Traditional ultrasound systems are costly, bulky, and cumbersome, making them impractical for routine use in most physical therapy or home settings.

These limitations highlight the need for more accessible and user-friendly approaches to ultrasound biofeedback. As noted by Stamm and Perotti [12], there is currently no wearable wireless device for physical therapy that transmits real-time ultrasound images of deep muscles to monitor stabilization exercises. The need for a smaller, portable ultrasound device that can be deployed quickly and inexpensively has been identified, consisting of a wireless transducer and mobile terminal that presents biofeedback in an easily understandable manner.

A previous study by Perotti et al. [7] investigated the needs and attitudes of older chronic back pain patients toward the ULTRAWEAR system—a mobile ultrasound system providing deep learning-based biofeedback on SSE execution. This research explored pain management behavior, experience with SSE, and requirements for the ULTRAWEAR system among 15 older adults with chronic back pain. The study found that patients demonstrated high willingness to use such a wearable ultrasound system both in physiotherapeutic practices and at home. Participants particularly valued the potential for automated detection and evaluation of muscle contraction states, which they perceived as a major advantage over traditional subjective feedback methods like palpation [7].

This previous work established that the system in development was perceived as a helpful solution to support learning about SSE. The researchers observed that the experts they interviewed desired a user interface (UI) that would be “playful and entertaining” to motivate and engage patients. However, some experts preferred a simple visualization based on common medical technology, while others suggested a design that could schematically illustrate the anatomical structure of the ultrasound image. What remained missing from this initial investigation was the perspective of patients with chronic back pain regarding these different visualization approaches. Recent reviews of geriatric digital health underline persistent barriers related to usability, engagement, and long-term adoption in older adults, supporting the need for user-centered and age-sensitive interface design [13,14].

Designing effective UI for older adults requires special consideration of age-related changes in visual perception, cognitive processing, and motor skills [15]. When designing feedback visualizations for physiotherapeutic applications, it is crucial to balance simplicity with informational content to ensure both ease of use and effectiveness [16]. In the context of the proposed system, the user interface (UI) serves three primary functions:

1. Providing real-time biofeedback on deep muscle activation;
2. Guiding the correct execution of segmental stabilization exercises;
3. Supporting adherence through motivational and educational elements.

While the UI itself does not directly reduce pain, it may contribute indirectly by improving exercise accuracy and long-term adherence.

Gamification can be defined as the use of game elements for the enhancement of user experience and engagement in contexts that are not games in themself, has emerged as a promising approach to increase motivation and adherence in health applications [17]. In the health context, gamification can motivate older people to use apps and help them achieve their health objectives [18].

Research on game element preferences in older adults shows mixed results. Koivisto & Malik [18] found in their review of gamification for older adults a variety of game elements, with the most common being (1) adaptive or increasing difficulty based on player progress, (2) social elements, (3) scores and points, (4) clear goals, and (5) progress indicators. Similarly, Sardi et al. [19] identified feedback, progression, rewards, and social interaction enhancement as the most relevant game elements for e-Health applications. Recent systematic reviews confirm that older adults remain underrepresented in gamification research, with most prior research targeting younger populations [20]. Similarly, a 2025 scoping review found persistent usability, self-efficacy, trust, and engagement barriers limiting digital health adoption among older adults, underscoring the need for age-sensitive UI and motivational design [13].

When designing gamified applications for older adults, it is essential to account for their specific needs and preferences. Koivisto and Malik [16] highlighted that older users often benefit from game elements that are intuitive, easy to understand, and tied to meaningful outcomes. Commonly effective elements include clear goals, adaptive difficulty, progress indicators, and social interaction features. Due to limited prior experience with digital games, game mechanics should be presented in a familiar and accessible manner to promote long-term engagement and support positive health-related behavior.

Kappen et al. [21] conducted an eight-week study examining how gamified applications can promote physical activity in adults aged 50 and above. Through thematic analysis of participant feedback, the study identified several motivational features that supported engagement: activity tracking for self-monitoring, goal-setting via simple challenges, reward mechanisms like badges, and clear feedback on progress. These features were found to enhance motivation and were interpreted within the framework of self-determination theory, highlighting the importance of autonomy, competence, and relatedness. While not formal design principles, these themes offer practical insights into effective gamification for older adults.

In addition to exercise feedback, pain education is an important component of comprehensive chronic back pain treatment. Pain Neuroscience Education (PNE) can improve knowledge and behavior of patients, thereby reducing pain intensity. Louw et al. [22] found strong evidence that pain education can change pain intensity, pain knowledge, disability, and pain behaviors in patients with musculoskeletal pain.

PNE aims to reduce pain and maladaptive behaviors by explaining to patients the underlying cognitive and biological principles of their pain experience. This often involves using pain analogies, educating patients about misconceptions about pain pathogenesis, and guiding them to make lifestyle and exercise changes. Misinterpreting pain on movement as harm leads to avoidance of movement; PNE works to change this perspective to the idea that pain serves as an alarm to protect the body [23].

The integration of pain education with exercise programs has shown promising results. However, as Airaksinen et al. [24] noted, chronic low back pain is unlikely to be effectively treated with a single intervention due to its multidimensional nature. Combined approaches that encourage activity, active self-management, and reduction in worry appear most effective. For older adults specifically, individualized education paired with appropriate exercises can provide a more comprehensive approach to pain management, addressing both the physical and psychological aspects of chronic pain.

Despite extensive research on chronic back pain treatment, ultrasound biofeedback, and gamification in health applications, several gaps remain in the literature. First, while ultrasound biofeedback has shown promise for improving SSE performance, existing systems are not optimized for user-friendly applications, particularly for older adults. Second, most gamification research focuses on younger populations, with limited understanding of how older adults perceive and respond to different game elements in health contexts. Third, although Perotti et al. [7] established the general acceptance of the ULTRAWEAR system, specific UI design preferences for visualizing ultrasound feedback in combination with pain education have not been investigated.

The current study addresses these gaps by examining which UI designs older adults with chronic back pain prefer for receiving ultrasound-based exercise feedback, which game elements they find most motivating, and whether they perceive value in integrating pain education into the application. While the present manuscript focuses on user requirements for interface design and motivation, the technical AI-based segmentation and real-time contraction-state assessment underlying the wearable ultrasound system are described in a separate publication by Strohm et al. [25], which reports automated segmentation for wearable ultrasound applications and real-time feasibility of contraction-state classification.

## 2. Materials and Methods

### 2.1. Study Design

The study employed an exploratory qualitative design, complemented by quantitative assessments to collect sociodemographic data from the participants with chronic back pain (CBP) and to assess the intention to use the different UI. Structured guided interviews were conducted to obtain insights into the evaluation of each UI version. In this report, we are adhering to the Consolidated Criteria for Reporting Qualitative Research (COREQ). Data analysis was performed using systematic qualitative content analysis, following the approach by Mayring [26]. Ethical approval was obtained from the Ethics Committee of Charité–Universitätsmedizin Berlin (approval number EA4/182/21). Additionally, the trial was registered in the German Clinical Trials Register (DRKS-ID: DRKS00026684; UTN: U1111-1271-0066).

### 2.2. Study Procedure

Participants with chronic back pain were recruited via email through the internal database of the Geriatrics Research Group at Charité–Universitätsmedizin Berlin. For participation in this study participants needed to be at least 65 years old, have experienced CBP for over six months, and be independently mobile. Exclusion criteria included cognitive impairments, sensory or motor deficits, inability to perform exercises while lying on the back, or severe spinal conditions such as tumors or fibromyalgia. Eligibility was determined through a telephone screening. Cognitive impairments were screened during the telephone interviews based on self-report and conversational assessment. However, no standardized cognitive assessment tool was applied, which may limit the detection of mild cognitive impairments. Prior to data collection, a pilot test was conducted with a 65-year-old woman without CBP to evaluate the clarity and feasibility of study materials and procedures. Based on her feedback, adjustments were made to the interview structure, including the reordering and removal of specific questions to improve coherence.

The study took place between November and December 2021 in the facility of the Geriatrics Research Group. We utilized consecutive sampling. Older adults with chronic back pain were first contacted through email, followed by a telephone screening. Out of 67 potential participants initially reached via email, 22 were screened over the phone. Among these, 22 older adults expressed interest and seven ultimately chose not to participate. Three of them declined without providing a reason, while another two were unable to attend due to falls that prevented travel to the study center. One individual did not show up for their scheduled appointment, and another canceled because of illness. All participants were exposed to all three UI designs (simple, anatomical, playful). The order of presentation was fixed. Recruitment was concluded after 15 interviews when initial theoretical saturation was achieved, as no new themes or codes emerged, indicating code saturation. This approach aligns with established qualitative methodology literature suggesting that thematic saturation in homogeneous samples is often reached within 12–15 interviews [27]. Each session lasted up to two hours. Three interviewers conducted the sessions: one male interviewer with expertise in public health as a member of the research group (I1), another male member of the research group with a physiotherapy background (I2) and a female working student specializing in human factors and design thinking (I3). Each interview involved both an interviewer and a second person taking notes to ensure accuracy in data collection.

For obtaining informed consent, the on-site study appointment included a detailed explanation of the study’s purpose, emphasizing the exploration of their expectations and preferences for a wearable system designed to provide real-time feedback on stabilization exercises via ultrasound. The use of AI to support the interpretation of the ultrasound images for both the patient and the physiotherapist was explained. The participants were informed about data confidentiality, pseudonymization procedures, and the recording process, with audio recordings deleted after transcription. Written informed consent was obtained from all participants, along with additional consent for the use of images and audio recordings. Following consent, participants completed baseline questionnaires, including demographic data and the Technology Usage Inventory (TUI) subscales on curiosity and anxiety. To ensure a shared understanding of stabilization exercises, a physiotherapist guided participants through two exercises: (1) an abdominal drawing-in maneuver to activate the transverse abdominis muscle, performed through controlled breathing and self-palpation, and (2) the same maneuver with alternating leg lifts. Verbal instructions and hands-on feedback helped participants recognize correct muscle engagement.

**Figure 1:**
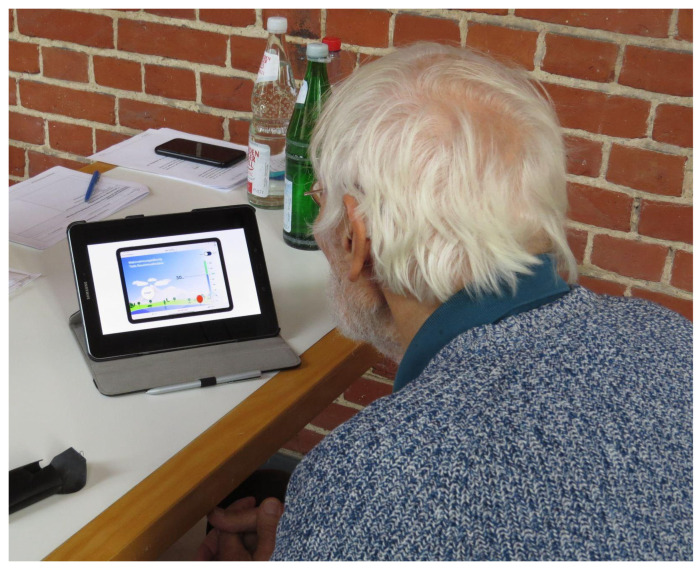
Demonstration of the potential UI designs to study participant.

Participants were then introduced to a potential ultrasound-based feedback system using a mockup device. Employing a Wizard-of-Oz method, a study staff member demonstrated the system by holding a non-functional transducer over their abdomen while a pre-recorded ultrasound video was displayed on a tablet. The anatomical structures were explained, and participants were encouraged to consider the potential benefits of real-time muscle visualization. Subsequently, a video demonstration of the prototype application was shown, followed by an evaluation of three different interface designs for training feedback (Figure 1). Participants provided feedback on each version, assessing aspects such as visual clarity, intuitiveness, and motivational impact. The qualitative semi-structured interview then focused on their overall impressions of the system, preferred interface elements, and additional design considerations for usability and engagement. To conclude, participants completed post-experiment TUI subscales measuring usability, skepticism, and intention to use the system. Additionally, they discussed potential enhancements, including alternative feedback modalities such as auditory cues, and their preferences for supplementary pain education content within the app.

### 2.3. Materials

#### 2.3.1. Technology Usage Inventory (TUI)

The Technology Usage Inventory (TUI) was used to evaluate participants’ acceptance and intentions towards the ULTRAWEAR system [28]. This validated instrument demonstrates strong reliability (Cronbach’s alpha ranging from 0.70 to 0.89) and consists of 30 items spread across nine distinct scales. These scales assess both pre-usage factors, such as curiosity and anxiety, and post-usage dimensions, including perceived interest, ease of use, usefulness, skepticism, accessibility, and intention to use. The pre-usage scales examine participants’ curiosity to explore new technologies and their anxiety about making mistakes or feeling overwhelmed, while post-usage scales focus on perceived utility, ease of interaction, and trust in the technology. The immersion scale was excluded in this study since our system does not incorporate immersive virtual environments. The intention to use (ITU) subscale utilizes a visual analog scale, where 0 represents “I agree” and 100 indicates “I do not agree.” The three items from this scale generate a score ranging from 0 to 300, with higher scores reflecting a stronger intention to use the technology. Participants filled out the questionnaire by indicating their level of agreement with a series of statements, with responses generally given on a seven-point Likert scale. For scoring a sum score is used. The original authors conducted a principal component analysis on an elderly subgroup, reporting moderate sampling adequacy (KMO = 0.67) and significant results from Bartlett’s test (*χ*^2^ (276) = 865.95; *p* < 0.001), confirming sufficient inter-item correlations. The internal consistency of the full TUI questionnaire was assessed across different age groups, with Cronbach’s alpha ranging from 0.66 to 0.89, demonstrating good reliability for the construct overall. We applied this assessment for the overall system and additionally for the three mockups used in this study. Here we assessed only the usability and intention to use subscales to assess these aspects separately for each mockup.

#### 2.3.2. Questionnaire for Sociodemographic Characteristics

A profile questionnaire was used to gather demographic information and to assess variables such as back pain location, pain intensity, quality of life impairment, and general attitude towards technology using the Affinity for Technology Interaction Scale [29]. This questionnaire provided relevant contextual data for participants, but additional questions outside the scope of the study were also included.

#### 2.3.3. UI Mockups and Design Prototypes

**Figure 2:**
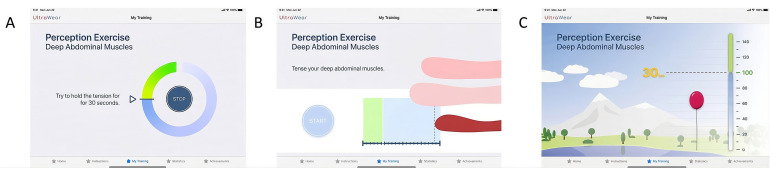
Translated UI design mockups; simple design (**A**), anatomical design (**B**), playful design (**C**).

To illustrate the app’s potential UI, a clickable mockup was created based on the findings of Stamm and Perotti [12], incorporating a simple, anatomical, and playful design. The mockup was developed by a partner specializing in UI design and was reviewed by experts in the development of user-friendly technology for older adults. A video was created to demonstrate the app’s features (Figure 2). The video journey includes an overview of the app’s educational content, feedback designs, game elements, and the diary feature.

The mockup incorporated three distinct feedback designs to simulate the ultrasound system: a simple, anatomical, and playful design.

- Simple Design (Figure 2A): This feedback system uses a clockwise-filling circle to represent deep muscle tension. The color of the circle shifts from blue (low tension) to green (optimal tension).
- Anatomical Design (Figure 2B): This design uses a visualization of muscles, where superficial muscles (rose) and deep muscles (red) move to indicate changes in muscle tension. Optimal tension is reached when the muscles move into the green area.
- Playful Design (Figure 2C): In this design, a balloon rises as muscle tension increases, reaching an optimal tension level when it crosses the green section of the scale.

Each feedback design provides the instruction “Hold the tension for 30 s” once optimal tension is achieved. In addition, several game elements, including points, badges, levels, and a leaderboard, were integrated to increase motivation for exercise. Participants’ preferences regarding these game elements were assessed during the study.

### 2.4. Additional Motivational Game Elements

**Figure 3:**
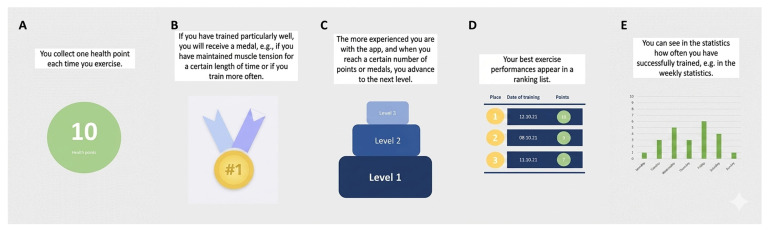
Translated mockups of the additional game elements; points (**A**), medals (**B**), leaderboard (**C**), levels (**D**), statistics (**E**).

Based on existing literature, common gamification elements such as points, badges, and levels were included to enhance motivation [30]. A chart was developed to track users’ progress across practice sessions, offering visual feedback on their performance. The game elements and progress chart were iteratively refined based on a pretest and further design development. While social interaction features were considered to enhance motivation, feedback from pretest interviews indicated that participants were not interested in sharing results with others. Therefore, the final system incorporated points, badges, levels, a leaderboard, and a progress chart, which were presented to participants through printed and laminated materials (Figure 3).

### 2.5. Data Collection and Analysis

All interviews were audio-recorded with participant consent and transcribed using f4transkript (dr. dresing & pehl GmbH, Marburg, Germany) following standardized transcription guidelines. A structured content analysis was conducted using Atlas.ti 8 (ATLAS.ti Scientific Software Development GmbH, Berlin, Germany), applying a combination of deductive and inductive coding approaches. Initially, a preliminary coding scheme was developed based on theoretical considerations and the interview guide. Subsequently, two researchers (I1 and I2) independently coded pilot interviews, refining the categorization system through an iterative review process. A final coding framework was then established, consisting of 22 codes organized into six overarching thematic categories. In total, 608 statements were coded across the 15 interviews.

To assess coding reliability, Krippendorff’s cu-alpha was calculated for both individual semantic domains and the dataset as a whole. In accordance with established standards, values exceeding α = 0.800 were considered reliable, while those between 0.667 and 0.800 were interpreted with caution. Any data with reliability below α = 0.667 was excluded from further statistical analysis.

Quantitative data analysis was performed using IBM SPSS Statistics 27 (IBM Corp., Armonk, NY, USA). Negatively coded items were reversed to ensure consistent interpretation of scale directionality, allowing higher scores to uniformly reflect more positive evaluations. Descriptive statistics were used to summarize participant characteristics, including age, pain severity, and quality-of-life impact. Categorical variables such as gender, back pain classification, pain location, and technology usage frequency were analyzed using relative distributions. Due to the sample size and non-normal distribution of data, standard deviations were omitted in favor of median and range reporting.

To assess perceptions of the app design, participants completed the Technology Usage Inventory (TUI), excluding the immersion scale. Individual item scores were summed within each scale, with negatively coded items reversed. The resulting scale scores were then averaged, facilitating comparisons with reference data from a previous validation study involving older adults (65+) [28].

To examine preferences for the three feedback designs (simple, anatomical, and playful), Friedman tests were conducted for the intention to use and ease of use scales. Given the ordinal nature of the data and the within-subject design, this non-parametric test was deemed appropriate. Post hoc pairwise comparisons were performed to determine significant differences between the design variants, and effect sizes were calculated to assess the strength of these differences.

## 3. Results

### 3.1. Research Participant Characteristics

15 older adults meeting the inclusion criteria participated in this study (Table 1). Detailed sample characteristics are presented in Table 1. The group included more women (53.3%) than men (46.7%). The average age for all participants was 75.67 years. All participants experienced low back pain in the lumbar spine, with the majority also reporting pain in additional areas.

### 3.2. Intercoder Reliability

The analysis of the intercoder reliability between the two coders were summarized and the various codes used for analysis were separated into three code groups. The following table (Table 2) lists these code groups used in the analysis of the qualitative interviews including the calculated Krippendorff’s cu-α. As seen in the table, the calculated intercoder reliability was above 0.800 in most code groups and even above 0.900 in one code group. In the two code groups “Prior experience with SSE” and “Usefulness and usage scenario of the future system”, the value was below 0.800 but above the threshold of 0.667. This could have been due to the very broad category in the code group “Usefulness and usage scenario of the future system” and the low number of citations in the code group “Prior experience with SSE”.

### 3.3. Feedback on the Design and Comprehensibility

The feedback on the system’s design and its comprehensibility was largely positive, with several participants highlighting the clear structure and ease of navigation. Seven participants emphasized that the system’s interface was intuitively organized, making it easy to use (“Well, first of all, it was really very pleasant. I didn’t feel overwhelmed at all. Sometimes you get programs where you think, oh my goodness, how am I supposed to figure this out? It had a playful approach, which, from my perspective, was very good—it wasn’t harsh but introduced things slowly. Somehow, I feel like I understood what it was supposed to be. It wasn’t one of those things where you’re left wondering, ‘What is this supposed to be?’ It was somehow understandable.” P05). The color scheme was also praised for being visually appealing and conducive to navigation. In particular, three participants noted that the system was well-suited for older users, citing its user-friendly menu navigation and clear presentation of content.

However, some critical points were raised. Two participants mentioned that the font size was too small, especially for older users, and expressed a need for clearer text formatting to enhance legibility (“It is quite strenuous. My eyes aren’t the best anyway. But you are targeting older people, after all.” P16). One participant remarked that the text was hard to read, particularly for those with impaired vision. Four participants also pointed out that the system tended to present too much information at once, which could potentially overwhelm users (“As I said, the information at the beginning is simply too much.” P06). These findings suggest that while the system’s overall design was seen as approachable, a focus on clearer text formatting and reducing the information load could improve usability.

Participants found the system’s design playful and cute, noting that it felt engaging without being overwhelming (“It’s cute how you’ve designed it—playful.” P03). Nonetheless, some felt that a stronger emphasis on essential elements and better prioritization of options could enhance its overall comprehensibility. One user suggested that, while the system was effective, certain features felt somewhat complex and difficult to understand without more in-depth explanations (“I assume that the relevant people have put thought into what is feasible and what it should achieve. I can imagine that. But, well, for me, it’s still a bit over my head at the moment.” P02).

### 3.4. Feedback on the Simple Design

The evaluation of the first mockup (“Simple Design”) revealed a mix of positive and critical feedback. Several participants appreciated the clear layout and intuitive interpretation of the interface, with two individuals specifically praising the logical structure that allowed them to complete tasks with minimal guidance. However, seven participants misunderstood the interpretation of the circle, mistakenly associating it with the time of contraction rather than the optimal achievable contraction (“Well, I understand that as the duration of the process.” P11). Three participants highlighted a lack of sufficient labeling or visual cues, creating difficulties in understanding certain functionalities (“What does it look like if I don’t do the exercise correctly? Does it not continue, or does it turn red? What currently looks so successful up there—green. Suppose I don’t do it right.” P16).

Interactivity was another area that received mixed feedback. Two participants noted that some interactive elements responded slower than expected, diminishing user satisfaction (“Yes. It’s getting slow now.” P15). Additionally, one participant suggested the addition of a step-by-step tutorial to guide first-time users more effectively (“But you need to have it properly explained.” P13). These responses indicate that while the design of the first mockup was functional, further adjustments in labeling, interactivity, and guidance could enhance the overall user experience.

### 3.5. Feedback on the Anatomical Design

The “Anatomical Design” was generally viewed more positively than the first mockup, but some areas for enhancement remained. Seven of the participants identified the red structures as muscles moving during the exercise while the other participants expressed irritation about this visualization. Four participants noted that the interface was more intuitive, with its design and smoother interactions aligning better with user expectations (“Yes, in detail, it’s better than the first one. It’s a bit more visualized, whether you’re in the green zone, whether you’re doing it right.” P09). Three participants appreciated the clearer visual design, particularly the improved organization of information and the use of better labels as well as the visualization of each muscle (“Hm, maybe it’s better than the first one because with the other one, it was the color that changed slowly, but here you can really see exactly how it slowly moves into the green and how far into the green, meaning how well you are doing it.” P09). However, a few participants still experienced difficulties with complex features that lacked adequate explanations (“You certainly need the explanation. But now that I’ve seen it, I can immediately categorize it.” P12). Two users suggested adding tooltips or brief pop-up explanations to clarify the purpose of more advanced features. Furthermore, three participants expressed a desire for greater customization options, such as the ability to adjust font sizes or color schemes to suit their personal preferences. Overall, the “Anatomical Design” was regarded positively in terms of responsiveness and visual clarity, but the need for more explanations was identified as an area for improvement.

### 3.6. Feedback on the Playful Design

The “Playful Design” was seen as an improvement to the first two mockups in terms of clarity and simplicity. Five participants praised the interface for being more engaging, fun, and straightforward, noting that the streamlined design helped reduce confusion compared to previous versions (“I mean, it certainly looks very funny, I would say. It’s not quite as technical. How should I put it (laughs)? It’s a bit simpler, I’d say, maybe easier to understand.” P02). Two participants specifically liked the inclusion of the balloon as a visual guide for the exercise, which contributed to a more engaging and understandable experience, describing it as cute and funny (“Well, such a balloon, something a bit cute.” P03). Despite these positive aspects, nine of the participants described the interface as inappropriate for senior users, stating it would be better suited for children (“Well, I would just bluntly say that for young people—I mean children—this thing might be the nicest.” P05). Three participants noted that elements such as button placement and color schemes varied between screens, disrupting the flow of the user experience (“It confuses me because it says 140 up there, and I can’t make sense of that.” P16).

### 3.7. User Preferences for Feedback Designs

Participants expressed differing opinions on the three feedback designs. Many liked the simple design for its clarity and ease of use, describing it as the most intuitive and straightforward option (“I think this is the best.” P08, “This is the most clear.” P18). Others preferred the anatomical design, seeing it as the most informative and medically relevant (“I think the anatomical one is the best.” P11, “I would prefer the anatomical one.”—P12). The gamified design received mixed feedback—some found it engaging, while others considered it too playful for a medical setting (“This is all too childish for me.”—P16, “I don’t like the playful approach.” P11). Several participants found it difficult to choose a clear favorite, as they saw all designs serving a similar purpose (“Basically, it all comes down to the same thing.”—P02). Some valued the ability to switch between designs depending on their mood or needs (“Depending on how you feel at the moment.” P05). Overall, preferences varied, with many emphasizing the importance of customization to accommodate individual user needs.

#### 3.7.1. Motivational Elements—Points

The participants provided varied feedback on the Points feature. Seven participants appreciated the feature, describing it as motivating and helpful in tracking their progress. The sense of accomplishment associated with earning points encouraged ongoing engagement with the system (“It would satisfy me somehow if I got points.” P04). However, one participant mentioned that the Points feature would resonate best with users if it were based on qualitative feedback on exercise performance (“So that means it gives me my exercises, and if they are good, I get many points; if they are bad, I get few points, to put it simply.” P05). Two participants pointed out that the criteria for awarding points were unclear, which reduced the perceived fairness and value of the system. To improve the system, participants suggested making the point-awarding criteria more transparent and offering goals to reach measured by points (“And then maybe there could be a target, like reaching ten points in a week. That wouldn’t be bad.” P05).

#### 3.7.2. Motivational Elements—Medals

The Medals feature garnered a range of responses. Four participants found the medals to be a valuable addition, offering recognition for reaching milestones and fostering a sense of achievement (“Yes, somehow an incentive, getting confirmation that you did well, is always a good thing.” P18). Some also appreciated the playful and engaging aspect that the medals brought to the system. However, three participants expressed concerns that the feature might seem superficial or unnecessary, particularly for those not interested in gamified elements (“Well, either the device is fun, and I notice the success. Fun is relative, but if I notice the success, then I’ll continue using it.” P13). One participant suggested offering more personalized rewards, such as custom medals or certificates, to make the feature more appealing.

#### 3.7.3. Motivational Elements—Leaderboard

The leaderboard feature received mixed feedback from participants. Four respondents appreciated it as a motivating element but noted that direct competition with other users was not necessary. They preferred comparing their exercise results with their own previous sessions (“I liked that you compete with yourself. You can also see progress.” P08). However, concerns were raised about its potential negative impact. Three participants mentioned that the leaderboard might discourage users who are not performing as well and therefore might not serve as a good motivational tool for older adults (“I think that might work for younger people. But I believe older individuals don’t need that anymore.” P04). To balance competition with inclusivity, it was recommended to implement alternative ranking systems, such as focusing on personal progress tracking rather than direct comparisons with others.

#### 3.7.4. Motivational Elements—Levels

The levels system was generally perceived as a positive motivational tool. Four participants highlighted its ability to provide a clear sense of progression, reinforcing their achievements and allowing them to assess their improvement over time. They noted that reaching new levels offered a tangible goal, which contributed to sustained engagement (“The levels are similar to many computer games, where you reach a new level.” P15). Two respondents suggested that progression should be tied to points earned through good exercise performance. Additionally, one participant proposed using descriptive names such as “beginner” and “expert” instead of numerical levels (P04). While the level system was considered beneficial in a clinical setting with a physiotherapist, it was seen as less necessary for home use (“Well, for professional use, for the physiotherapist, it might be quite interesting. But for me as a patient and for at home, I wouldn’t necessarily need it.” P17). Another participant recommended aligning the levels with the difficulty or pacing of exercises to match individual preferences or experience (“But you could also introduce different exercises that are more challenging.” P08).

#### 3.7.5. Motivational Elements—Statistics

The statistics feature was generally well-received, with fourteen participants highlighting its value in providing detailed insights and a simple overview of their progress. One participant emphasized that the statistics would be a good way to receive longitudinal feedback on exercise performance (“You had that statistic at the end. I would be interested in that. But afterwards, not in parallel.” P03). These insights were seen as empowering and motivating, particularly when represented visually through charts or graphs. The absence of gamified elements was especially described as appropriate (“Yes, for example, that is also a good thing. In my opinion, this is no longer playful or gimmicky; it feels more serious.” P05). One participant suggested offering a simplified view of key metrics, such as a monthly summary, for users who prefer a more basic overview.

#### 3.7.6. Suggestions for Additional System Feedback—Visual

Most participants praised the use of additional visual feedback as an effective and intuitive method for conveying system responses. Only one participant considered additional visual feedback unnecessary. Six respondents specifically noted that clear visual cues, such as color-coded notifications, progress bars, and animations, helped reduce cognitive load and facilitated understanding of the system’s status or required actions. Four participants suggested displaying the actual ultrasound image of the muscles between exercises to enhance comprehension of anatomical structures, emphasizing the need for thorough instruction beforehand (“And here, with ultrasound, you first need a trained eye. You certainly need an explanation. But now that I’ve seen it, I can immediately understand it. So I think both are important, at least that’s what I would say.” P12). Additionally, it was mentioned that incorporating instructional videos could support the use of the system in home settings (“Yes, as a video, perhaps showing someone demonstrating it. And you would need to follow along within a certain time frame.” P12). Another participant suggested that additional visual support could introduce an element of fun to the training, proposing that humorous elements could also be integrated (“A joke is always good, laughing, lightening things up a bit.” P08).

#### 3.7.7. Suggestions for Additional System Feedback—Acoustic

Participants had mixed reactions to the possibility of adding additional acoustic feedback options to the system. Three respondents found it useful for confirming the start or end of one exercise or drawing attention to important notifications. It was mentioned that acoustic information would be especially helpful when users lying on their back would be unable to look at the screen (“That would certainly be a nice feature if it were not only visual but also audible. So that you can see that you don’t always have to stare at it. When I tense this, I have to keep looking.” P15). Several participants specifically preferred spoken instructions over simple sounds, as they felt a voice gave the impression of personal guidance and support (“I prefer speech. Think of people my age, 76, 80, 85; they would also prefer speech.” P10). An acoustic signal via speech was also considered as helpful when providing instructions on exercise execution (“Maybe the lady could say something, either you see it, but she could also say, for example, Contract now,’ ‘Hold for 30 s.’ And then I can see whether I decrease during those 30 s or not, so it would be useful to say something because I don’t always have to look here. That would be stressful.” P12). Two participants mentioned that frequent or loud audio signals could become distracting, particularly during prolonged usage. One participant suggested that auditory alerts should be reserved for critical moments, such as signaling incorrect exercise execution or urgent updates, rather than routine confirmations. The type and quality of sound feedback were also discussed. Participants expressed a preference for low, harmonic tones rather than high-pitched signals, which they found unpleasant or disruptive. Additionally, they suggested varying the pitch or intensity of the tones based on muscle tension, providing dynamic feedback that adapts to user performance (“Rising tones, something like the beginning, and then it would get higher or louder, or something like that.” P15). Some users also highlighted the importance of sound feedback for scenarios where the screen isn’t visible, such as exercising in the dark (“You are not a patient, as a subject or as a perceiver, and much could be done through sound. It could be used in the dark, perhaps, or while lying down after getting up or first thing in the morning. And again at night before going to bed, much could be done with sound, I think.” P11).

### 3.8. Psychoeducation

The possibility of incorporating informational material on pain and pain management into the system was generally well received, with 10 participants finding it a helpful addition. They emphasized that the materials should be useful, clear and informative and could help inexperienced users (“For me specifically, where I am a bit of a layperson in this regard, it would be quite useful.” P02). Participants particularly appreciated content on anatomy, muscle structure, and pain management, as well as practical information on treatment options, everyday tips, and additional exercises (“Yes, for example, dealing with pain and how back pain arises, that would be good to be able to retrieve; that wouldn’t be bad.” P05). Several users also suggested periodic updates on new medical research and new types of exercises and evolving best practices for pain management (“Well, if scientifically and medically new exercises were included. Where you could say, it has now been shown to be very helpful. Then you could expand it with these exercises.” P09). Five respondents found psychoeducation as unnecessary within the system, since they would get the information needed by the therapist.

A recurring suggestion was to present information in different formats based on complexity. Basic topics could be provided as text with images, while more complex subjects (e.g., advanced pain mechanisms) would be better suited for video explanations (“So, if you make a video, you could also nicely overlay it with a graphic, that wouldn’t be a problem today. If you only do text, yes, not everyone just reads text. With video, you have both image and text. The learning effect is also greater, normally. There are others who learn better through text, so why not both? The effort is minimal.” P10). Three participants expressed that passive learning through long text sections was less effective than interactive learning elements, such as step-by-step tutorials or scenario-based guidance. Accessibility was another key consideration. Two participants stressed the need to provide content in multiple formats, including text, audio, and video, to accommodate different learning styles and sensory impairments. One participant specifically recommended the inclusion of subtitles in all video materials to ensure comprehension for users with hearing difficulties. Finally, one participant suggested integrating psychoeducation in the form of an always available portal, allowing users to access only needed information at will “Portals, you can always check in because they are constantly updated. You’re the one who has already experienced a lot, done a lot, seen a lot. When you check in on such a portal, if something new appears, is it something for me? Can I take something from it? I find that useful.” P06).

### 3.9. Results of the Technology Usage Inventory

The results of the Technology Usage Inventory provide a comprehensive overview of participants’ acceptance and intended adoption of the ULTRAWEAR system. The sample exhibited moderate levels of curiosity (*M* = 18.13, *SD* = 4.87) and relatively low anxiety (*M* = 12.67, *SD* = 6.69) regarding new technologies, indicating a generally favorable predisposition prior to system interaction. Post-usage evaluations revealed a balanced degree of interest (*M* = 17.40, *SD* = 7.91) and a positive perception of ease of use (*M* = 17.93, *SD* = 3.22). The high mean score for perceived usefulness (*M* = 22.67, *SD* = 3.89) underscores the strong functional value attributed to the system by participants. Moreover, skepticism toward the technology was notably low (*M* = 5.27, *SD* = 2.09), suggesting robust trust in the system’s reliability and performance. The accessibility score (*M* = 14.20, *SD* = 3.78) indicates moderate perceptions of the system’s ease of access and usability. The intention to use the score was high (*M* = 260.67, *SD* = 29.10) on a scale ranging from 0 to 300, indicating a stated willingness to use the system. However, intention to use does not necessarily translate into actual long-term usage in real-world settings. These findings indicate a generally positive perception of the system among participants. However, given the exploratory nature of the study and the small, potentially technology-affine sample, these results should be interpreted cautiously and do not necessarily translate into real-world adoption.

### 3.10. Comparison of the Ease of Use and Intention to Use Between the Three Mockup Designs

User friendliness did not differ between the different UI versions (Table 3). However, significant differences in the intention to use subscales (*χ*^2^ (2) = 8.40, *p* = 0.015) were present. Kendall’s W was 0.28, indicating a medium effect size. Subsequently, the Wilcoxon rank sum test was conducted for paired samples to analyze the differences in the ITU subscale between the three mockups. Here, only the differences between the playful mockup and the anatomical mockup (*Z* = −2.05, *p* = 0.041) and between the playful mockup and the simple mockup (*Z* = −2.95, *p* = 0.003) were significant, while the values for the playful design were lower in each case. The effect sizes in both comparisons were a large effect (playful/anatomical: *r* = −0.53; playful/simple: *r* = −0.76).

## 4. Discussion

This study demonstrated that the ULTRAWEAR system and the different UI versions were generally well-received. Participants favored the simple and anatomical designs for receiving feedback on deep muscle activation during exercise. Additionally, game elements such as progress tracking through charts and points were perceived as potentially enhancing motivation, suggesting they could be beneficial for older adults with chronic back pain. Integrating a level system to track progress may further encourage sustained use. There is also evidence that incorporating pain education would be well received by patients and could support patient engagement and promote physical activity.

When comparing the three feedback designs, older adults with chronic low back pain most frequently mentioned the simple and anatomical UI as the preferred method of receiving exercise feedback using the ultrasound-based wearable. A significant difference was observed in the intention to use ratings, with the simple and anatomical designs preferred over the gamified version. This suggests that a highly playful design may not align with the preferences of older adults with back pain. One potential reason could be that overly colorful and childlike esthetics may feel inappropriate in a medical or rehabilitation context, as patients might perceive such designs as lacking seriousness. The concept of infantilization—where older adults are treated in a childlike manner—is often viewed as disrespectful and can lead to resistance [31]. Supporting this, participants appreciated the simple design for its professional appearance, while the anatomical design was valued for providing additional insights into muscle activity. It should be noted that the preference for simple and anatomical interfaces may partly reflect the medical and rehabilitative context, where participants expected a ‘serious’ and professional appearance, rather than age alone. In other contexts (e.g., leisure activities), older adults might evaluate playful elements differently; thus, our findings should not be generalized to all digital applications for this age group.

All three feedback designs were considered easy to understand, with the simple version rated as the most intuitive, followed by the anatomical and gamified designs. However, the scale representation in the simple and anatomical designs was found to be unclear, while fewer participants reported issues with the playful design. This could be attributed to the order in which the designs were presented, as familiarity with the interface might have improved comprehension. Importantly, no significant differences in ease of use were found among the feedback designs, indicating that design preferences were likely influenced by other factors rather than usability alone. In the literature, factors such as icon size, density of information and color contrast are seen as UI elements influencing usability for older adults [32,33]. Additionally, participants expressed a strong desire for audio feedback, supporting the idea that combining visual and auditory cues could improve user experience. According to Wickens’ [34] multiple resources theory, dual-task performance—such as monitoring feedback while engaging in muscle activation—benefits from distributing cognitive load across different sensory modalities. This aligns with common physiotherapy practices, where verbal feedback is used to guide muscle activation [35,36].

Overall, the app was rated highly in terms of usability and acceptance. The intention to use the system was high, suggesting strong user interest in the ultrasound-based wearable. While many older adults perceive new technology as beneficial for health purposes, this positive attitude is often linked to a perceived usefulness and ease of use of the technology [37,38,39,40]. Participants found the app well-structured, easy to understand, and visually appealing. The accessibility of the system was rated slightly above average, though some uncertainty remained regarding the cost of hardware for personal or clinical use. This might be even more prominent in future applications when the price for the wearable may be foreseeable and the application in home-settings might be feasible. The older adults who participated in this study may have had a generally positive attitude towards technology (as indicated in the Affinity for Technology Interaction score). Prior research has identified two primary personas among older adults using gamified health apps—those hesitant toward technology and those more accepting [41]. The current findings suggest that app appearance can influence engagement, supporting the idea that customization and adaptation of design elements could improve adoption among diverse user groups.

Game elements that motivated participants most were charts, followed by points and levels. Progress tracking is a well-established motivator in gamified health applications, especially for older users [18,42]. While points are commonly used in gamified apps, leaderboards and badges were less favored [30]. This may be because leaderboards often encourage direct competition, which older users do not find appealing, which was also evident in our study. Prior research suggests that older adults over 75 prefer collaborative rather than competitive game elements, aligning with findings that leaderboards and badges in this study were less favored [38]. Furthermore, participants generally criticized as too gamified UI, consistent with Tizuka et al.’s [41] recommendation to avoid overwhelming older users with excessive gamification which can even.

Beyond visualized game elements, participants identified additional motivational factors, with achievement emerging as the strongest. This aligns with the idea that individuals with chronic pain may be particularly driven by the goal of improving mobility and reducing discomfort. Social interaction, whether with peers or physiotherapists, was also highlighted as an important motivator. Prior studies emphasize the role of social engagement in increasing adherence to health apps [18]. Other motivating factors included clear goal setting, reminders, and exercise variety, all of which are commonly recommended in gamified health interventions [43,44].

Although our study did not directly measure adherence or health-economic outcomes, the observed preference for simple, intuitive, and medically credible interfaces may be relevant for sustained use, which is a prerequisite for clinical benefit. This is consistent with guideline-based chronic low back pain care emphasizing exercise, education, and self-management, and with broader evidence that wearables can support chronic disease management, although the evidence for cost-effectiveness remains limited and mixed [45,46,47].

Finally, participants expressed interest in receiving pain education within the app, though they did not consider it a necessity. Previous research suggests that combining pain education with physical activity can lead to long-term improvements in pain intensity, disability, and overall quality of life [48]. While prior studies have tested various pain education models, findings remain mixed. Ryan et al. [49] found that pain education alone reduced chronic low back pain more effectively than when combined with stabilization exercises, whereas Frost et al. [50] reported the opposite effect when using a different educational approach. Given that participants in the present study preferred pain education as a complement to stabilization exercises, an app-based education module could focus on key topics such as anatomy, pain mechanisms, and posture correction. However, the limited number of studies specifically addressing pain education for older adults with back pain highlights the need for further research in this area [51].

### 4.1. Strength and Limitations

A key strength of our study is its mixed-methods approach, which combines qualitative insights with quantitative baseline assessments to provide a comprehensive evaluation of the ultrasound-based wearable system for older adults with chronic back pain. The use of structured guided interviews, systematic qualitative content analysis, and validated measurement tools, such as the Technology Usage Inventory (TUI), enhances the reliability and depth of the findings. Additionally, the inclusion of a diverse range of feedback designs and motivational game elements allowed for a nuanced understanding of user preferences. The study also benefits from a rigorous recruitment and screening process, ensuring that participants met well-defined inclusion criteria, and from adherence to established reporting guidelines (COREQ). However, several limitations should be noted. The sample size was relatively small (15 participants) and limited to older adults with a generally positive attitude toward technology, which may limit the generalizability of the findings to a broader population. Additionally, the Wizard-of-Oz methodology, while useful for early-stage user testing, does not fully replicate real-world system functionality, potentially influencing participant perceptions. We also acknowledge that a non-randomized order of UI presentation may have introduced learning or familiarity effects and thus biased the evaluation. The study was also conducted in a controlled environment rather than home settings, which may not fully capture real-life usability challenges. Lastly, while initial theoretical saturation was achieved, further research with larger and more diverse samples is needed to validate the effectiveness and long-term acceptance of the proposed system. Finally, high intention-to-use scores, as measured with the TUI, may not directly translate into real-world adoption, especially in older adults who face additional practical barriers (e.g., access to devices, support, comorbidities). Our findings should thus be interpreted as an indication of perceived acceptability under controlled conditions.

### 4.2. Conclusions

This study provides evidence that older adults with chronic back pain are receptive to an ultrasound-based wearable app, with strong preference for simple and anatomical feedback designs. Key motivating game elements include charts, points, and levels, while overly playful or competitive features may be less effective. Additionally, auditory feedback and optional pain education could enhance engagement. While these findings are promising, a further RCT is needed to validate the effectiveness of these design choices in larger and more diverse populations.

### 4.3. Practical Implications

The results of this study highlight several practical implications for the development of digital health applications. First, system design should prioritize clarity and ease of use, ensuring that navigation and interface elements are intuitive, especially for older users. Second, feedback mechanisms should be customizable to accommodate individual preferences regarding visual and acoustic cues. This could enhance user comfort while maintaining the effectiveness of real-time feedback.

Third, gamification features should be carefully implemented, with an emphasis on personalization. Providing users with the option to enable or disable gamification elements based on their preferences may improve overall acceptance and motivation. Lastly, psychoeducational content should be interactive and accessible, incorporating multiple formats such as text, audio, and video to cater to different learning preferences.

Fourth, our gamification findings are based on self-reported preferences, perceived motivation, and intention-to-use scores, rather than on objective behavioral data such as long-term usage or adherence. As perceived usefulness and intention do not always translate into actual behavior, future longitudinal studies should evaluate how these UI and gamification elements influence real-world use and exercise adherence.

Although the present study did not measure adherence or economic outcomes directly, improved usability and acceptance are plausible prerequisites for sustained use, which is necessary before any downstream effects on rehabilitation adherence or service efficiency can be expected. Improved usability and engagement may increase adherence to spinal stabilization exercises, which is a key challenge in conservative chronic back pain management among older adults. In the long term, wearable biofeedback systems may support home-based rehabilitation and reduce dependence on supervised therapy sessions, potentially lowering healthcare utilization and associated costs.

## Figures and Tables

**Table 1:** Study sample characteristics.

Sociodemographic Data	Sample
Number of participants [*n*]	15
Sex (Female/Male) [in %]	53.33/46.66
Age [M (SD) in years]	75.67 (5.04)
Age range [min.–max. in years]	69–85
Highest educational attainment [in %]	
University	40
Advanced technical college certificate	40
High school	6.66
Secondary school	6.66
Main school	6.66
Marital status [in %]	
single	6.66
married	66.66
divorced	0
widowed	26.66
cohabiting	0
Associated diagnoses of chronic pain [in %]	
Unspecified LBP	33.33
Disk herniation	20
Spinal stenosis	13.33
Scoliosis	13.33
Affinity for Technology Interaction Scale *M* (*SD*)	3.5 (1.75)

**Table 2:** Code groups used for the analysis and calculated intercoder reliability.

Code Group	Calculated Krippendorff’s cu-α
Semantic domain “UI evaluation”	0.80
Codes: General evaluation of the UI Positive evaluation UI 1 Irritations UI 1 Improvement ideas UI 1 Positive evaluation UI 2 Irritations UI 2 Improvement ideas UI 2 Positive evaluation UI 3 Irritations UI 3 Improvement ideas UI 3 Preferred UI Additional acoustic feedback Additional visual feedback	
Semantic domain “UI elements for training motivation”	0.97
Codes: Motivational element—points Motivational element—medals Motivational element—leaderboard Motivational element—statistics Motivational element—levels Motivational element—preference	
Semantic domain “Psychoeducation”	0.84
Codes: Psychoeducation—general attitude Psychoeducation—format Psychoeducation—content	

**Table 3:** TUI subscale comparison between UI versions.

TUI Scale	Simple Design	Anatomical Design	Playful Design
	*M* [*SD*], 95%-CI UB, LB	*M* [*SD*], 95%-CI UB, LB	*M* [*SD*], 95%-CI UB, LB
Ease of use	13.40 [2.87], 11.81, 14.99	14.47 [0.83], 14.01, 14.93	13.93 [1.75], 12.96, 14.90
Intention to use	209.00 [90.56], 158.85, 259.15	245.87 [40.66], 223.35, 268.39	144.80 [101.42], 88.64, 200.96

CI = confidence interval, UB = upper bound, LB = lower bound.

## Data Availability

The dataset underlying this study is available upon request from the corresponding author.

## References

[1] Von Der Lippe E., Krause L., Prost M., Wengler A., Leddin J., Müller A., Zeisler M.-L., Anton A., Rommel A. (2021). BURDEN 2020 Study Group Prävalenz von Rücken- und Nackenschmerzen in Deutschland. Ergebnisse der Krankheitslast-Studie BURDEN 2020. J. Health Monit..

[2] Rackwitz B., de Bie R., Limm H., von Garnier K., Ewert T., Stucki G. (2006). Segmental Stabilizing Exercises and Low Back Pain. What Is the Evidence? A Systematic Review of Randomized Controlled Trials. Clin. Rehabil..

[3] Hodges P.W. (2003). Core Stability Exercise in Chronic Low Back Pain. Orthop. Clin..

[4] Hides J., Stanton W., Dilani Mendis M., Sexton M. (2011). The Relationship of Transversus Abdominis and Lumbar Multifidus Clinical Muscle Tests in Patients with Chronic Low Back Pain. Man. Ther..

[5] Richardson C., Hodges P.W., Hides J., Richardson C. (2004). Therapeutic Exercise for Lumbopelvic Stabilization: A Motor Control Approach for the Treatment and Prevention of Low Back Pain.

[6] Coulombe B.J., Games K.E., Neil E.R., Eberman L.E. (2017). Core Stability Exercise Versus General Exercise for Chronic Low Back Pain. J. Athl. Train..

[7] Perotti L., Stamm O., Mesletzky L., Vorwerg S., Fournelle M., Müller-Werdan U. (2023). Needs and Attitudes of Older Chronic Back Pain Patients towards a Wearable for Ultrasound Biofeedback during Stabilization Exercises: A Qualitative Analysis. Int. J. Environ. Res. Public Health.

[8] Valentín-Mazarracin I., Nogaledo-Martín M., López-de-Uralde-Villanueva I., Fernández-de-las-Peñas C., Stokes M., Arias-Buría J.L., Díaz-Arribas M.J., Plaza-Manzano G. (2021). Reproducibility and Concurrent Validity of Manual Palpation with Rehabilitative Ultrasound Imaging for Assessing Deep Abdominal Muscle Activity: Analysis with Preferential Ratios. Diagnostics.

[9] Henry S.M., Teyhen D.S. (2007). Ultrasound Imaging as a Feedback Tool in the Rehabilitation of Trunk Muscle Dysfunction for People with Low Back Pain. J. Orthop. Sports Phys. Ther..

[10] Lin S., Zhu B., Zheng Y., Huang G., Zeng Q., Wang C. (2021). Effect of Real-Time Ultrasound Imaging for Biofeedback on Trunk Muscle Contraction in Healthy Subjects: A Preliminary Study. BMC Musculoskelet. Disord..

[11] Henry S.M., Westervelt K.C. (2005). The Use of Real-Time Ultrasound Feedback in Teaching Abdominal Hollowing Exercises to Healthy Subjects. J. Orthop. Sports Phys. Ther..

[12] Stamm O., Perotti L., Duffy V.G., Gao Q., Zhou J., Antona M., Stephanidis C. (2022). Expert Requirements for an Ultrasound-Based Wearable Using Deep Learning for Exercise Feedback in Older Chronic Back Pain Patients. Proceedings of the HCI International 2022—Late Breaking Papers: HCI for Health, Well-Being, Universal Access and Healthy Aging.

[13] Birati Y., Tzemah-Shahar R. (2026). Barriers to Digital Health Adoption in Older Adults: Scoping Review Informed by Innovation Resistance Theory. J. Med. Internet Res..

[14] Isaradech N., Sirikul W. (2025). Digital Health Tools Applications in Frail Older Adults—A Review Article. Front. Digit. Health.

[15] Mayer C., Morandell M., Gira M., Sili M., Petzold M., Fagel S., Schüler C., Bobeth J., Schmehl S., Stephanidis C., Antona M. (2013). User Interfaces for Older Adults. Universal Access in Human-Computer Interaction. User and Context Diversity.

[16] Barandas M., Gamboa H., Fonseca J.M. (2015). A Real Time Biofeedback System Using Visual User Interface for Physical Rehabilitation. Procedia Manuf..

[17] Deterding S., Dixon D., Khaled R., Nacke L. (2011). From Game Design Elements to Gamefulness: Defining “Gamification”. Proceedings of the 15th International Academic MindTrek Conference: Envisioning Future Media Environments, Tampere Finland, 28–30 September 2011.

[18] Koivisto J., Malik A. (2021). Gamification for Older Adults: A Systematic Literature Review. Gerontol..

[19] Sardi L., Idri A., Fernández-Alemán J.L. (2017). A Systematic Review of Gamification in E-Health. J. Biomed. Inform..

[20] Hong W., Choi M. (2026). The evolving landscape of gamification for older adults: Evidence from a systematic review. Gerontechnology.

[21] Kappen D.L., Mirza-Babaei P., Nacke L.E. (2020). Technology Facilitates Physical Activity Through Gamification: A Thematic Analysis of an 8-Week Study. Front. Comput. Sci..

[22] Louw A., Diener I., Butler D.S., Puentedura E.J. (2011). The Effect of Neuroscience Education on Pain, Disability, Anxiety, and Stress in Chronic Musculoskeletal Pain. Arch. Phys. Med. Rehabil..

[23] Wood L., Hendrick P.A. (2019). A Systematic Review and Meta-analysis of Pain Neuroscience Education for Chronic Low Back Pain: Short-and Long-term Outcomes of Pain and Disability. Eur. J. Pain.

[24] Airaksinen O., Brox J.I., Cedraschi C., Hildebrandt J., Klaber-Moffett J., Kovacs F., Mannion A.F., Reis S., Staal J.B., Ursin H. (2006). Chapter 4European Guidelinesfor the Management of Chronicnonspecific Low Back Pain. Eur. Spine J..

[25] Strohm H., Rothluebbers S., Perotti L., Stamm O., Fournelle M., Jenne J., Guenther M. (2024). Contraction Assessment of Abdominal Muscles Using Automated Segmentation Designed for Wearable Ultrasound Applications. Int. J. Comput. Assist. Radiol. Surg..

[26] Mayring P., Bikner-Ahsbahs A., Knipping C., Presmeg N. (2015). Qualitative Content Analysis: Theoretical Background and Procedures. Approaches to Qualitative Research in Mathematics Education: Examples of Methodology and Methods.

[27] Guest G., Namey E., Chen M. (2020). A Simple Method to Assess and Report Thematic Saturation in Qualitative Research. PLoS ONE.

[28] Kothgassner O.D., Felnhofer A., Hauk N., Kastenhofer E., Gomm J., Kryspin-Exner I. (2013). Technology Usage Inventory (TUI): Manual.

[29] Franke T., Attig C., Wessel D. (2019). A Personal Resource for Technology Interaction: Development and Validation of the Affinity for Technology Interaction (ATI) Scale. Int. J. Hum. Comput. Interact..

[30] Cechetti N.P., Biduki D., De Marchi A.C.B. (2017). Gamification Strategies for Mobile Device Applications: A Systematic Review. Proceedings of the 2017 12th Iberian Conference on Information Systems and Technologies (CISTI), Lisbon, Portugal, 21–24 June 2017.

[31] Salari S.M. (2005). Infantilization as Elder Mistreatment: Evidence from Five Adult Day Centers. J. Elder Abus. Negl..

[32] Zhou C., Yuan F., Huang T., Zhang Y., Kaner J. (2022). The Impact of Interface Design Element Features on Task Performance in Older Adults: Evidence from Eye-Tracking and EEG Signals. Int. J. Environ. Res. Public Health.

[33] Zhang X., Dolah J. (2024). A Study of Key Factors Influencing the Usability of Smartphone Graphical User Interfaces for Older Adults. PaperASIA.

[34] Wickens C.D. (2008). Multiple Resources and Mental Workload. Hum. Factors.

[35] Staker J.L., Evans A.J., Jacobs L.E., Ebert T.P., Fessler N.A., Saini G., Ludewig P.M. (2022). The Effect of Tactile and Verbal Guidance during Scapulothoracic Exercises: An EMG and Kinematic Investigation. J. Electromyogr. Kinesiol..

[36] Jones S.A., Pamukoff D.N., Mauntel T.C., Blackburn J.T., Myers J.B. (2018). The Influence of Verbal and Tactile Feedback on Electromyographic Amplitude of the Shoulder Musculature During Common Therapeutic Exercises. J. Sport Rehabil..

[37] Williams S., Orsega-Smith E., Ruggiero L. (2020). Exploring Technology Perceptions and Intentions to Use in Older Adults. Innov. Aging.

[38] Moxley J., Sharit J., Czaja S.J. (2022). The Factors Influencing Older Adults’ Decisions Surrounding Adoption of Technology: Quantitative Experimental Study. JMIR Aging.

[39] Mitzner T.L., Boron J.B., Fausset C.B., Adams A.E., Charness N., Czaja S.J., Dijkstra K., Fisk A.D., Rogers W.A., Sharit J. (2010). Older Adults Talk Technology: Technology Usage and Attitudes. Comput. Hum. Behav..

[40] Abdelrahman N.G., Haque R., Polverento M.E., Wendling A., Goetz C.M., Arnetz B.B. (2020). Brain Health: Attitudes towards Technology Adoption in Older Adults. Healthcare.

[41] Tizuka M.M., Clua E.W.G., De Castro Salgado L.C. (2020). Investigating M-Health Gamification Rewards Elements for Adults 50+. Proceedings of the 2020 IEEE 8th International Conference on Serious Games and Applications for Health (SeGAH), Vancouver, BC, Canada, 12–14 August 2020.

[42] Liu Y., Alexandrova T., Nakajima T. (2011). Gamifying Intelligent Environments. Proceedings of the 2011 international ACM Workshop on Ubiquitous Meta User Interfaces, Scottsdale AZ, USA, 1 December 2011.

[43] Cechetti N.P., Bellei E.A., Biduski D., Rodriguez J.P.M., Roman M.K., De Marchi A.C.B. (2019). Developing and Implementing a Gamification Method to Improve User Engagement: A Case Study with an m-Health Application for Hypertension Monitoring. Telemat. Inform..

[44] Kappen D.L., Mirza-Babaei P., Nacke L.E. (2017). Gamification through the Application of Motivational Affordances for Physical Activity Technology. Proceedings of the Annual Symposium on Computer-Human Interaction in Play, Amsterdam, The Netherlands, 15–18 October 2017.

[45] Overview. Chronic Pain (Primary and Secondary) in over 16s: Assessment of All Chronic Pain and Management of Chronic Primary Pain. Guidance. NICE. https://www.nice.org.uk/guidance/ng193.

[46] Overview. Low Back Pain and Sciatica in over 16s: Assessment and Management. Guidance. NICE. https://www.nice.org.uk/guidance/ng59.

[47] Mattison G., Canfell O., Forrester D., Dobbins C., Smith D., Töyräs J., Sullivan C. (2022). The Influence of Wearables on Health Care Outcomes in Chronic Disease: Systematic Review. J. Med. Internet Res..

[48] Galán-Martín M.A., Montero-Cuadrado F., Lluch-Girbes E., Coca-López M.C., Mayo-Iscar A., Cuesta-Vargas A. (2019). Pain Neuroscience Education and Physical Exercise for Patients with Chronic Spinal Pain in Primary Healthcare: A Randomised Trial Protocol. BMC Musculoskelet. Disord..

[49] Ryan C.G., Gray H.G., Newton M., Granat M.H. (2010). Pain Biology Education and Exercise Classes Compared to Pain Biology Education Alone for Individuals with Chronic Low Back Pain: A Pilot Randomised Controlled Trial. Man. Ther..

[50] Frost H., Moffett J.A.K., Moser J.S., Fairbank J.C.T. (1995). Randomised Controlled Trial for Evaluation of Fitness Programme for Patients with Chronic Low Back Pain. BMJ.

[51] Zahari Z., Ishak A., Justine M. (2020). The Effectiveness of Patient Education in Improving Pain, Disability and Quality of Life among Older People with Low Back Pain: A Systematic Review. J. Back Musculoskelet. Rehabil..

